# Nutritional Composition, Physicochemical Properties, Antioxidant Activity, and Sensory Quality of *Matricaria chamomilla*-Enriched Wheat Bread

**DOI:** 10.3390/foods14050838

**Published:** 2025-02-28

**Authors:** Khawla Kerbab, Ibtissem Sanah, Fairouze Djeghim, Nadjah Belattar, Valentina Santoro, Maria D’Elia, Luca Rastrelli

**Affiliations:** 1 Laboratoire de Génie Biologique Valorisation et Innovation des Produits Agroalimentaires Institut ISTA-Ain M’Lila, Université Larbi Ben M’hidi Oum El-Bouaghi, Oum El-Bouaghi 04000, Algeria; kerbab.khawla@univ-oeb.dz (K.K.); sanah.ibtissem@univ-oeb.dz (I.S.); 2 Unité de Recherche Valorisation des Ressources Naturelles, Molécules Bioactives et Analyses Physico Chimiques et Biologiques (VARENBIOMOL), Université Constantine 1, Route de Aïn El Bey, Constantine 25017, Algeria; nadjahorg@gmail.com; 3 Laboratoire de Recherche en Sciences Alimentaires, Formulation, Innovation, Valorisation et Intelligence Art Ficielle (SAFIVIA), Institut de la Nutrition, de l’Alimentation et des Technologies Agro-Alimentaires (INATAA), Université Frères Mentouri Constantine 1, Constantine 25017, Algeria; 4 Équipe FNPAA, Laboratoire de Nutrition et Technologie Alimentaire (L.N.T.A), Institut de la Nutrition, de l’Alimentation et des Technologies Agro-Alimentaires (INATAA), Université Frères Mentouri Constantine 1, Constantine 25017, Algeria; fairouze.djeghim@umc.edu.dz; 5 Department of Pharmacy, University of Salerno, Via Giovanni Paolo II, 132, 84084 Fisciano, Italy; vsantoro@unisa.it (V.S.); mdelia@unisa.it (M.D.); 6 National Biodiversity Future Center—NBFC, 90133 Palermo, Italy; 7 Dipartimento di Scienze della Terra e del Mare, University of Palermo, 90135 Palermo, Italy

**Keywords:** *Matricaria chamomilla*, fortified bread, antioxidant activity, UPLC/MS-MS analysis, sensory evaluation, plant-based by-products

## Abstract

This study evaluates the effects of fortifying bread with different concentrations (3%, 10%, and 30%) of *Matricaria chamomilla* L. (MC) infusion and powder, derived from the plant’s aerial parts (stem, leaf, and flower). UPLC/MS-MS analysis of MC infusion and powder ethanolic extract confirmed the presence of polyphenolic compounds, including flavonoids, contributing to enhanced antioxidant and enzyme inhibitory properties. The physicochemical, antioxidant, and sensory properties of the enriched breads were assessed. Hierarchical cluster analysis revealed that breads enriched with 30% MC powder (BP-MC 30%) and infusion (BI-MC 30%) exhibited superior overall quality compared with other formulations. The enriched breads showed increased protein, fiber, and antioxidant content. Specifically, BI-MC 30% demonstrated superior antioxidant activity, while BP-MC 30% provided the highest fiber content. Sensory evaluation indicated that the enriched breads retained sensory properties similar to those of the control bread, despite the nutritional improvements. These findings suggest that incorporating *Matricaria chamomilla*, particularly at 30%, enhances the nutritional profile and antioxidant properties of bread while maintaining sensory characteristics close to those of traditional bread. This research highlights the potential of using chamomile’s aerial parts in the valorization of plant-based by-products for functional bakery product development.

## 1. Introduction

Bread is a staple food consumed globally, providing a significant contribution to daily nutrient intake. It accounts for over 10% of the daily intake of several essential nutrients, such as proteins, thiamine, niacin, folate, iron, zinc, copper, and magnesium [1]. Furthermore, bread supplies approximately 32% of daily caloric intake and 36% of protein requirements in urban households [2]. Revered as the “staff of life”, bread holds deep cultural and social significance, symbolizing nourishment and community across diverse societies [3,4]. The World Health Organization (WHO) recommends a daily intake of 200–250 g of bread, underscoring its importance as a key component of the human diet [5,6]. Despite its long history of over 10,000 years, bread consumption has declined in some regions worldwide, with average daily intake in certain areas now reduced to just 2–3 slices per person [1,7].

This decline, coupled with increasing consumer demand for healthier food options, has sparked interest in reformulating bread to enhance its nutritional profile. Consumers are increasingly seeking products with reduced fat, sugar, and sodium contents, while also favoring those with added functional ingredients that provide health benefits [8,9,10]. Although bread is sometimes perceived negatively due to its association with weight gain and health concerns, its nutritional value is well-documented and continues to make it a valuable dietary staple [11,12]. Therefore, there is a growing need to innovate and fortify bread with natural, functional ingredients that improve both its nutritional composition and sensory characteristics.

In recent years, the incorporation of plant-based components into bread formulations has shown great promise in enhancing its nutritional content, particularly in terms of fiber, protein, and antioxidant activity. These functional ingredients not only address dietary deficiencies, but also cater to the increasing demand for healthier, functional foods. One such promising ingredient is *Matricaria chamomilla* L. (chamomile), a well-known medicinal herb with a rich history of use in folk medicine, particularly in Algeria. Chamomile is a potent source of bioactive compounds, including polyphenols (coumarins and flavonoids), sesquiterpenes, and essential oils [13,14]. Chamomile contains high levels of both phenolic acids and flavonoids, which contribute to its notable antioxidant properties [15,16]. Given its antioxidant richness, chamomile is an ideal candidate for fortifying functional foods, including bread, to improve their nutritional value.

In addition to its nutritional benefits, chamomile presents an opportunity for the valorization of plant-based by-products. Traditionally, chamomile flower heads (capitula) are harvested to produce commercial infusions or teas, often leaving the rest of the plant—comprising stems, leaves, and flowers—underutilized. This study proposes a more sustainable approach by utilizing the entire aerial part of the plant, including the leaves, stems, and flowers, as a functional ingredient in bread. By doing so, we not only enhance the nutritional profile of bread, but also reduce waste by giving a second life to these by-products, which would otherwise be discarded. A portion of chamomile crops could be specifically cultivated and harvested for bread production, employing both the flowers and the aerial parts, thus creating an integrated value chain that benefits both the food industry and the environment.

In this study, we aimed to develop an innovative bread product enriched with chamomile in two distinct forms: an infusion and a powder. The bread was prepared using a basic formulation of whole wheat flour, yeast, salt, and water, which was then fortified with chamomile at concentrations of 3%, 10%, and 30%. Two bread formulations were developed: one enriched with chamomile powder (BP-Mc) and the other with chamomile infusion (BI-Mc). To characterize the bioactive compounds present in chamomile, we employed UPLC/MS-MS analysis to provide detailed profiling of the phytochemicals in the extracts. The prepared dough was fermented and baked according to standard procedures, with a control bread produced using the same methodology, but without the addition of chamomile. The focus of this research was to evaluate the physicochemical properties, antioxidant capacity, and sensory qualities of the enriched bread, with the aim of assessing the potential of chamomile as a natural functional ingredient in improving bread’s nutritional profile and consumer acceptability.

## 2. Materials and Methods

### 2.1. Plant Material

*Matricaria chamomilla* L. (chamomile, *babounj*) was collected in 2019 from the Sétif region in the eastern part of Algeria. A voucher specimen has been deposited at the Herbarium of the VARENBIOMOL Research Unit, University of Constantine 1. The plant material, including the leaves and flowers, was air-dried at ambient temperature to preserve the bioactive compounds.

### 2.2. Reagents and Solvents

Analytical-grade ethanol (EtOH), methanol (MeOH), chloroform, and MS-grade formic acid (HCOOH) were obtained from Merck Chemicals (Milan, Italy). MS-grade acetonitrile and water were purchased from Romil (Cambridge, UK). Ultrapure water (18 MΩ) was prepared using a Milli-Q purification system (Millipore, Bedford, TX, USA).

All chemicals and reagents used in this study were of analytical grade and were sourced from commercial suppliers. The Folin–Ciocalteu reagent, gallic acid (used for calibration), quercetin (used as the standard for flavonoid content), 1,1-diphenyl-2-picrylhydrazyl (DPPH), 2,2′-azino-bis(3-ethylbenzothiazoline-6-sulfonic acid) (ABTS), butylated hydroxytoluene (BHT), butylated hydroxyanisole (BHA), acarbose, and the copper(II) reduction assay (CUPRAC) reagent were obtained from Sigma-Aldrich (St. Louis, MO, USA).

Ethanol (analytical-grade) was used for the ethanolic extraction of *Matricaria chamomilla*, and distilled water was used for the preparation of solutions and infusions. All reagents were used without further purification and stored according to the manufacturer’s instructions. Glassware was cleaned and sterilized before use.

### 2.3. Extraction Procedure

The chamomile infusion was prepared by adding 250 g of air-dried leaves and flowers to 500 mL of boiling distilled water. The mixture was then steeped for 10 min. The infusion was subsequently filtered through filter paper (0.45 µm) to remove solid residues. The water content of the filtered infusion was then removed under vacuum at 40 °C using a rotary evaporator (B690, BUCHI Italia srl, Milan, Italy) to yield the chamomile infusion extract (Mc-I), with a final weight of 4.14 g. The chamomile powder was prepared by grinding 50 g of air-dried leaves and flowers first in a mortar and then in a coffee grinder, yielding 44.07 g of fine chamomile powder (Mc-P). From this plant material, the ethanol extract was prepared by extracting 10 g of the aerial parts with a 7:3 ethanol–water solution. The mixture was filtered through a 0.45 µm filter paper, and the solvents were removed under vacuum at 40 °C using a rotary evaporator. The bread was extracted in the same way: 2 g of the sample was mixed with 40 mL of 70% ethanol/water and stirred magnetically at room temperature for 6 h. The resulting mixture was filtered, and the residue was re-extracted under the same conditions. After the second extraction, the solutions were combined, evaporated at 40 °C in a rotary evaporator, lyophilized, and stored at −20 °C until further analysis.

### 2.4. Total Phenolic Content

The total polyphenol content in the chamomile infusion, extract, and enriched bread sample formulations was determined using the Folin–Ciocalteu method [17]. To 1 mL of each sample, 1 mL of Folin–Ciocalteu reagent was added. After 3 min, 1 mL of 25% sodium carbonate solution was added. The mixture was incubated for 2 h at room temperature. The samples were then centrifuged at 4000 rpm for 4 min, and the absorbance was measured at 670 nm using a spectrophotometer. The total phenolic content is expressed as milligrams of gallic acid equivalents per gram of dry extract (mg GAE/g) (Figure S1).

### 2.5. Total Flavonoid Content

The total flavonoid content in the chamomile infusion, extract, and enriched bread samples was quantified using a spectrophotometric method based on the formation of a flavonoid–aluminum complex [18]. To 1 mL of sample solution, 1 mL of 2% AlCl_3_ methanol solution was added and incubated for 30 min at room temperature. The absorbance was measured at 430 nm. Quercetin was used as the standard, and the total flavonoid content was expressed as quercetin equivalents (QEs) per gram of dry extract (Figure S2).

### 2.6. UHPLC-PDA-ESI-HRMS Characterization of Matricaria Ingredients

The phytochemical profile of *Matricaria chamomilla* extract was analyzed using a Vanquish Flex UHPLC system coupled to a dual detector setup: a diode array detector (DAD) and an Orbitrap Exploris 120 mass spectrometer with a heated electrospray ionization source (HESI-II, ThermoFisher Scientific, Milano, Italy). Chromatographic separation was achieved on a Kinetex C18 column (100 × 2.1 mm I.D., 2.6 μm; Phenomenex, Bologna, Italy) with a binary gradient of water (A) and acetonitrile (B), both containing 0.1% formic acid. The gradient program was as follows: 0–3 min, 2% B; 3–6.5 min, 2–9% B; 6.5–11.5 min, 9% B; 11.5–15 min, 9–30% B; 15–17 min, 30–98% B; and 17–19 min, 98% B. UV spectra were collected between 200 and 600 nm, and high-resolution mass spectrometry (HRMS) data were acquired in both positive and negative ionization modes. MS data were obtained in the full MS/dd-MS2 acquisition mode with resolutions of 60,000 and 30,000 WHM, respectively. Fragmentation was induced by stepped collision energy (HCD: 20, 40, and 60). Compound identification was carried out by integrating UV spectra data and HRMS/MS, supplemented by literature references. This approach enabled the identification of 41 specialized metabolites in the *Matricaria chamomilla* infusion and 37 in the ethanolic extract, mainly phenolic acids and flavonoids.

### 2.7. Determination of Antioxidant Activity

ABTS assay: the antioxidant activity of the chamomile infusion and extract was evaluated using the ABTS (2,2′-azino-bis(3-ethylbenzothiazoline-6-sulfonic acid)) radical cation decolorization assay. The ABTS radical was generated by reacting ABTS (7 mM) with potassium persulfate (2.45 mM) in water and allowing the reaction mixture to stand at room temperature for 12–16 h. The radical solution was then diluted with ethanol to an absorbance of approximately 0.70 at 734 nm. A total of 10 µL of each sample was added to 990 µL of ABTS solution, and the absorbance was measured after 6 min at 734 nm. The antioxidant activity was quantified as the percentage of ABTS radical scavenging relative to a control. For the controls, BHT and BHA were used at concentrations of 0.1 mM and 0.5 mM, respectively, to compare the antioxidant efficacy [19].

DPPH Assay: The scavenging activity of free radicals in the chamomile infusion, extract, and enriched bread samples was evaluated using the 1,1-diphenyl-2-picrylhydrazyl (DPPH) radical, as described by Sharma and Bhat [20], with minor modifications. A methanolic solution of DPPH (0.11 mM) was prepared by dissolving 4 mg of DPPH in 100 mL of methanol. To 1 mL of each sample at a given concentration, 1 mL of the DPPH solution was added. The absorbance was measured at 517 nm after 30 min of incubation in the dark. Three replicates were performed for each sample concentration. Ascorbic acid was used as the standard antioxidant.

The antioxidant activity, related to the scavenging effect of the DPPH radical, is expressed as the percentage inhibition (PI), calculated using the following Formula (1):

(1) $$\%PI=\frac{\left(AControl-ATest\right)}{AControl}\times 100$$

The results are expressed as the IC50 values (mg/mL), which correspond to the concentration required for 50% inhibition. BHT and BHA were included as positive controls at concentrations of 0.1 mM and 0.5 mM, respectively.

CUPRAC assay: The CUPRAC (CUPric Reducing Antioxidant Capacity) assay was used to measure the antioxidant activity of *Matricaria chamomilla* (MC) extracts and infusions. The assay was carried out according to established protocols with slight modifications. A 96-well microplate format was utilized to evaluate the antioxidant potential at various concentrations (3.125 µg/mL to 200 µg/mL) for both the MC ethanol extract (EtOH) and infusion. This method relies on measuring the absorbance of the Cu(I)-neocuproine (Nc) complex formed through the redox reaction between chain-breaking antioxidants and the CUPRAC reagent, Cu(II)-Nc, with absorbance recorded at the peak absorption wavelength of 450 nm [21]. The absorbance readings were compared with standard antioxidant compounds, such as BHA (butylated hydroxyanisole) and BHT (butylated hydroxytoluene), which served as reference compounds. The antioxidant activity was quantified by calculating the absorbance at each concentration, and the results were used to determine the IC50 value, representing the concentration required to inhibit 50% of the CUPRAC reaction.

### 2.8. Measurement of α-Amylase Inhibitory Activity

The α-amylase inhibitory activity was evaluated using the iodine/potassium iodide (IKI) method, based on Randhir and Shetty [22] with slight modifications. The assay was conducted in a 96-well microplate reader, with each well containing a volume of 250 µL. The reagents used included α-amylase (1 U/mL in phosphate buffer, pH 6.9, with 6 mM NaCl), a 0.1% starch solution (prepared by heating 0.1 g of starch in 100 mL of water with stirring), 1 M HCl (prepared by mixing 4.17 mL of concentrated HCl with 45.83 mL of distilled water), a 5 mM iodine–potassium iodide (IKI) solution (prepared by dissolving 3 g of KI and 0.125 g of iodine in 100 mL of distilled water), and phosphate buffer (pH 6.9 with 6 mM NaCl, made by dissolving 35 mg of NaCl in 100 mL of buffer solution). In the procedure, 25 µL of the sample (extract or infusion) was combined with 50 µL of α-amylase solution and incubated for 10 min at 37 °C. After this, 50 µL of the starch solution was added and the incubation continued for another 10 min at 37 °C. The reaction was stopped by adding 25 µL of 1 M HCl, followed by 100 µL of the IKI solution. The absorbance was measured at 630 nm. Acarbose was used as the standard. The percentage of inhibition was calculated by comparing the absorbance values for the sample, blank, and control.

### 2.9. Bread Production

Whole wheat flour (100% whole grain, non-GMO) used for bread preparation was procured from a local Algerian market. The flour had a moisture content of ≤15.5% and an ash content of ≥1.20%. Instant dry yeast (Saf-Instant, Marcq-en-Barœul, France) and salt were sourced locally as well.

### 2.10. Bread Enrichment and Formulation

The bread was prepared using a basic formulation consisting of 100 g of whole wheat flour, 2 g of salt, 4 g of yeast, and 60 mL of water. The dough was enriched with chamomile at concentrations of 3%, 10%, and 30% in two distinct formulations. The first formulation was enriched with chamomile powder (Mc-P), and the second formulation was enriched with chamomile infusion (Mc-I). The chamomile powder and infusion were added to the basic dough mixture at these concentrations to create the two experimental formulations.

The dough was thoroughly mixed until the ingredients were evenly distributed. The prepared dough was placed in designated molds and allowed to ferment at 40 °C for 35 min. After fermentation, the dough was baked in a preheated electric oven (Memmert D39263/D39264, Schwabach, Germany) at 260 °C ± 10 °C for 25 min A control bread was prepared without the addition of chamomile (neither powder nor infusion), using the same basic recipe and procedure described above, to serve as a baseline for comparison in subsequent analyses.

### 2.11. Analytical Composition

The analyses were conducted using standard methods as described in the AOAC guidelines [23]. Moisture content was determined by slicing and powdering the samples in a blender, followed by drying them in an oven at 130 °C until a constant weight was achieved, according to the AOAC 934.06 method. The total protein content was determined by the Kjeldahl method using a Kjeldahl apparatus, with a conversion factor of 6.25. The total lipid content was determined by the Soxhlet extraction method, following the AOAC 991.43 method. The crude fiber content was quantified using the Weende method (AFNOR NF V03-40 1993). The carbohydrate content was determined using a differential calculation method. The ash content was determined by incinerating the sample at 550 °C, following the AOAC 923.03 method.

### 2.12. Bread Quality Measurement

The effects of the chamomile plant material and its extracts on bread quality were assessed by measuring specific volume, weight loss, pH, crust and crumb color, and crumb cell structure in the control bread and the BI-MC 30%- and BIPMC 30%-enriched breads. The weight and volume of the bread were measured 1 h after baking. The volume was determined using the rapeseed displacement method (AACC 10.05, 2000) [24]. The specific volume (cm^3^/g) was calculated as the ratio of the bread volume to its weight. Weight loss was calculated as the percentage difference between the weight of the dough before fermentation and the weight of the baked bread, using Formula (2):

(2) $$\%\;weight\;loss=\frac{Dough\;weight-Bread\;weight}{Dough\;weight}\times 100$$

For pH measurement 10 g of bread sample was ground and mixed with 100 mL of distilled water. After 30 min of shaking with an orbital shaker (Heidolph Polymax 1040, Schwabach, Germany), the pH of the supernatant was measured after 10 min of settling (Majzoobi et al., 2017). Crust and crumb color were measured using the Color Grab app (version 3.6.1, Loomatix Ltd., Munchen, Germany) following the method of Djeghim et al. [25]. A controlled lighting environment was created using a polystyrene box with a 1.2 W 5 V white LED light to ensure uniform illumination. The CIE-Lab* color space model was employed to measure lightness (L*) and chromaticity (a* for green to red, and b* for blue to yellow). The crumb structure was analyzed by slicing the bread into 1 cm thick sections, capturing images in TIFF format, and processing them using ImageJ software (version 1.43u, Wayne Rasband, NIH, Bethesda, MD, USA). The images were cropped to focus on the crumb, converted to 8 bit grayscale, and analyzed for cell number (cells/mm^2^), average cell size, area fraction, perimeter, circularity, and solidity.

### 2.13. Sensory Evaluation of the Bread

Sensory evaluation was conducted using a 7-point hedonic scale (1 = dislike extremely, 7 = like extremely (Figure S3)), with ten trained panelists (five males and five females, aged 20–40 years). The panelists evaluated the bread for color, aroma, taste, volume, alveolation, texture (crispness and hardness), and overall acceptability. Samples were presented in a randomized order with a 3-digit code. The evaluation took place 2 h post-baking, with panelists instructed to cleanse their palates with water between samples.

### 2.14. Statistical Analysis

Statistical analysis was conducted using JMP Pro 17.0.0 software (SAS Institute Inc., Cary, NC, USA, 2021). All measurements were performed in triplicate, and data were expressed as means ± standard deviation. One-way analysis of variance (ANOVA) was performed to determine significant differences between means for each variable. Pairwise comparisons were performed using Tukey’s Honest Significant Difference (HSD) test, with significance set at *p* ≤ 0.05. To assess the relationships and groupings between bread samples based on their physicochemical composition, antioxidant activity, and sensory attributes, a heatmap was generated using hierarchical cluster analysis (HCA). Ward’s method and squared Euclidean distance were used to cluster the data. Control bread was not included in this cluster analysis. The HCA results were visualized graphically in the form of a dendrogram and heatmap to illustrate the similarities and differences among the bread formulations (BI-MC 3%, BI-MC 10%, BI-MC 30%, BP-MC 3%, BP-MC 10%, BP-MC 30%).

## 3. Results

To explore the potential for bread enrichment, we chose to use chamomile infusion and powdered chamomile, rather than the ethanolic extract, to assess their practical applicability in the bread matrix. The primary reason for this decision was to evaluate how these more accessible forms of chamomile can be incorporated into bread in a cost-effective and convenient manner while potentially delivering similar bioactive effects to those observed with the ethanolic extract. The ethanolic extract, however, was used to characterize the composition and functional potential of *Matricaria chamomile*, providing insight into its bioactive compounds.

### 3.1. Phytochemical Profiling of Matricaria chamomilla Infusion and Ethanolic Extract

**Figure 1:**
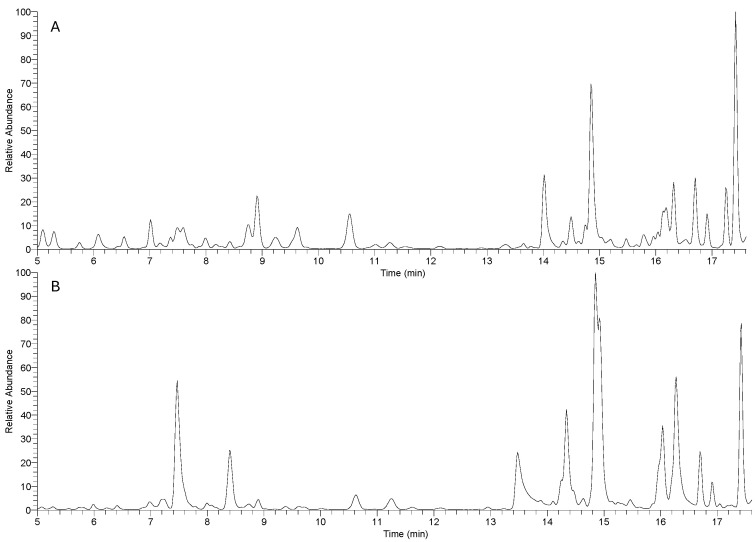
Profile of *Matricaria chamomilla* extract: UHPLC-HRMS chromatogram of infusion (**A**) and ethanolic extract (**B**).

The phytochemical profiles of the *Matricaria chamomilla* infusion and ethanolic extract were examined using UHPLC-HRMS, identifying several specialized metabolites, particularly phenolic acids and flavonoids. In total, 41 compounds were identified in the infusion, while 37 were detected in the ethanolic extract, suggesting subtle but meaningful differences between the two preparation methods. In addition, the ethanolic extract-enriched bread (BE-MC) was analyzed to assess the retention of polyphenolic compounds following baking. Figure 1 presents the UHPLC-HRMS chromatograms for both the chamomile infusion (A) and ethanolic extract (B), showcasing their distinct chemical compositions. The chromatogram of the infusion (A) reveals several distinct peaks with relatively short retention times, corresponding to hydrophilic polyphenolic compounds, such as caffeoylquinic acid derivatives. These peaks suggest the efficient extraction of water-soluble phenolic acids, known for their bioactive properties, including antioxidant effects. In contrast, the chromatogram for the ethanolic extract (B) displays a broader range of peaks with longer retention times, primarily representing other polyphenolic compounds, such as flavonoid glycosides and caffeic acid derivatives. The higher intensity and wider distribution of these peaks indicate that ethanol is more effective in extracting these polyphenols, which are less soluble in water and tend to elute later. These chromatographic differences, characterized by both peak intensity and retention time, highlight the distinct bioactive profiles of the two preparations, underscoring how the choice of solvent influences the extraction of polyphenolic compounds. Table 1 reports the main compounds identified in the infusion, ethanolic extract, and enriched bread, respectively. One notable difference between the infusion and ethanolic extract is the presence of certain compounds in one sample over the other. For instance, in the infusion (Table 1), we identified several caffeoylquinic acid derivatives, such as caffeoyl quinic acid (MC_1) and caffeoyl quinic hexoside (MC_4), which are key metabolites frequently found in *Matricaria chamomilla* [14,16]. These compounds were also present in the ethanolic extract, but with differing intensities. Interestingly, some caffeic acid derivatives (MC_11 and MC_14) showed slightly higher concentrations in the ethanolic extract, which might be attributable to the solvent’s ability to extract more polar metabolites from the plant matrix compared with water.

Flavonoids, including luteolin (MC_35) and apigenin (MC_36), were identified in both the infusion and ethanolic extract, but certain flavonoid glycosides (e.g., apigenin O-diglucuronide, MC_24, and luteolin O-caffeoylhexoside (MC_35), were more prominent in the ethanolic extract. This could indicate that the ethanolic extraction method is more efficient for isolating flavonoid derivatives, which are typically less water-soluble. On the other hand, the infusion seems to better retain certain hydrophilic compounds, such as hydroxyjasmonic acid (MC_19), which were identified at higher concentrations compared with the ethanolic extract [26]. Polyphenolic compounds like dicaffeoylquinic acid (MC_28 and MC_30) were abundant in both samples. The compounds detected in the MC-E-enriched bread were found to be largely consistent with those identified in the ethanolic extract of chamomile, confirming the possibility of evaluating the quality of the chamomile-enriched bakery products based on the UHPLC-HRMS method developed.

### 3.2. Antioxidant Activity, Total Polyphenols, and Flavonoids of Matricaria chamomilla Infusion and Ethanolic Extract

The antioxidant activities of both the chamomile infusion and ethanolic extract were evaluated using three distinct assays: the DPPH (2,2-diphenyl-1-picrylhydrazyl) assay, the ABTS (2,2′-azino-bis(3-ethylbenzothiazoline-6-sulfonic acid)) assay, and the CUPRAC assay. BHT (butylated hydroxytoluene) and BHA (butylated hydroxyanisole) were used as positive controls for both assays.

#### 3.2.1. DPPH Assay

The results of the DPPH assay showed a clear difference in the antioxidant activity between the two chamomile preparations. The ethanolic extract demonstrated a higher antioxidant potential, with the IC50 value calculated at 13.15 ± 0.95 µg/mL (Table 2), indicating a stronger ability to scavenge free radicals compared with the chamomile infusion. At the highest tested concentration of 800 µg/mL, the ethanolic extract achieved an inhibition of 88.42 ± 0.22% (Table S1), which is consistent with its higher polyphenol and flavonoid content. On the other hand, the chamomile infusion, with an IC50 of 24.46 ± 0.35 µg/mL, showed a slightly lower inhibition, with a maximum inhibition of 87.15 ± 0.30% at the same concentration of 800 µg/mL.

#### 3.2.2. ABTS Assay

The results from the ABTS assay further reinforce the superior antioxidant properties of the ethanolic extract compared with the chamomile infusion. At concentrations ranging from 12.5 to 800 µg/mL (Table S2), the ethanolic extract demonstrated an increasing inhibition percentage, reaching 78.87 ± 0.66% at the highest concentration, with an IC50 value of 81.04 ± 0.42 µg/mL. The chamomile infusion still offered substantial antioxidant potential, with a maximum inhibition of 79.88 ± 0.39% at 800 µg/mL and an IC50 of 93.91 ± 0.94 µg/mL (Table 2), The control substances, BHT and BHA, demonstrated superior antioxidant activities with inhibitions of 92% and 89%, respectively, highlighting the stronger free radical scavenging ability of synthetic antioxidants when compared with chamomile preparations.

#### 3.2.3. CUPRAC Assay

The antioxidant activity of MC extracts and infusions was evaluated through the CUPRAC assay. For both the ethanol extract and infusion, there was a dose-dependent increase in absorbance with higher concentrations, indicating increased antioxidant activity. The MC ethanol extract showed absorbance values ranging from 0.33 ± 0.01 at 3.125 µg/mL to 3.02 ± 0.07 at 200 µg/mL (Table S3). The IC50 value for the MC ethanol extract was 4.95 ± 0.14 µg/mL. Similarly, the MC infusion displayed absorbance values from 0.37 ± 0.01 at 3.125 µg/mL to 3.29 ± 0.20 at 200 µg/mL, with an IC50 value of 4.54 ± 0.11 µg/mL (Table 2).

#### 3.2.4. Total Polyphenols and Flavonoids

The total polyphenol and flavonoid contents were quantified to evaluate the bioactive components of the chamomile infusion and ethanolic extract (Table 2). The total phenolic content (TPC) of the ethanolic extract was significantly higher at 110.32 ± 0.62 µg GAE/mg compared with the chamomile infusion, which showed a TPC of 82.82 ± 0.42 µg GAE/mg. This difference can be attributed to the greater solubility of polyphenolic compounds in ethanol, facilitating a more efficient extraction process. Similarly, the total flavonoid content (TFC), quantified through a colorimetric assay with aluminum chloride and expressed as quercetin equivalents (QEs), was significantly higher in the ethanolic extract, which contained 89.79 ± 0.59 µg QE/mg, compared with the chamomile infusion, which had 47.92 ± 0.29 µg QE/mg of total flavonoids.

### 3.3. α-Amylase Inhibitory Activity

The α-amylase inhibitory activity of both the ethanolic extract and infusion of chamomile was assessed at different concentrations (Tables S4 and S5). The ethanolic extract showed a dose-dependent inhibition of α-amylase, with an IC50 value of 21.32 ± 0.74 µg/mL. The inhibition ranged from 42.47 ± 0.23% at the lowest concentration (1.9531 µg/mL) to 58.11 ± 0.15% at the highest concentration (125 µg/mL). On the other hand, the chamomile infusion demonstrated an IC50 value of 11.27 ± 1.15 µg/mL, with inhibition varying from 44.41 ± 0.19% at 1.9531 µg/mL to 57.96 ± 0.27% at 125 µg/mL. The chamomile infusion exhibited stronger α-amylase inhibitory activity compared with the ethanolic extract, as indicated by the lower IC50 value (11.27 ± 1.15 µg/mL vs. 21.32 ± 0.74 µg/mL) (Table 2).

### 3.4. Bread Production and Characteristics

The bread formulations were produced by fortifying a basic dough with *Matricaria chamomilla* L. (MC) in two forms: powder (BP-Mc) and infusion (BI-Mc) at concentrations of 3%, 10%, and 30%. The fortifying process resulted in significant differences in the physicochemical properties of the breads, with the enriched formulations generally exhibiting enhanced nutritional content compared with the control bread. The incorporation of chamomile, particularly at higher concentrations, influenced key characteristics, such as the protein, fiber, and antioxidant levels, as well as sensory attributes including texture, aroma, and overall acceptability.

Enriched breads had a lower moisture content compared with the control bread, with BI-MC 30% showing the lowest moisture level (12.55%), followed by BP-MC 30% (30.74%).

The protein content was significantly higher in the MC-enriched breads, with BI-MC showing the highest value (14.78%), followed by BP-MC (12.06%). The lipid content was slightly lower in the enriched breads (0.8% for BI-MC and 1.2% for BP-MC) compared with the control bread (1.8%). BP-MC bread exhibited the highest fiber content (10.6%), reflecting the fiber-rich nature of chamomile. BI-MC showed the highest carbohydrate content (59.46%), while BP-MC and control breads had lower carbohydrate contents (39.23% and 38.44%, respectively). Both BI-MC and BP-MC showed elevated ash contents, with BI-MC at 8.48% and BP-MC at 6.17%, compared with the control bread (4.33%). These differences highlight how the incorporation of chamomile, especially at higher concentrations, modifies the composition of the bread, enhancing its nutritional profile (Table 3 and Table S6).

### 3.5. Bread Quality Evaluation

During the fermentation process, the different bread formulations, including those enriched with *Matricaria chamomilla* (MC), showed a significant increase in volume. This increase is visible in Figure S3, which shows the bread during the leavening phase before baking. However, after baking, marked differences were observed in the physicochemical and sensory characteristics of the breads, especially for those enriched with chamomile at 30% (both powder and infusion), which showed noticeable differences compared with the control bread (Table 4), as illustrated in Figure S4. These results are consistent with those in the literature on bread with plant-based fortifications [27,28,29,30,31,32,33].

#### 3.5.1. Specific Volume

Breads enriched with 30% chamomile (both powder and infusion forms) had significantly lower specific volumes compared with the control bread, with values of 2.19 ± 0.07 cm^3^/g for BI-MC 30% and 2.26 ± 0.20 cm^3^/g for BP-MC 30%, compared with the control’s 3.36 ± 0.001 cm^3^/g (*p*-value = 0.004).

#### 3.5.2. Weight Loss

There were no significant differences in weight loss among the bread formulations, with values ranging from 30.04 ± 0.84% (BI-MC) to 32.69 ± 1.32% (BP-MC), compared with the control (31.30 ± 0.95%).

#### 3.5.3. pH

The pH of the 30% chamomile infusion bread (BI-MC 30%) and chamomile powder bread (BP-MC 30%) was significantly lower than that of the control, with pH values of 5.30 ± 0.02 and 5.43 ± 0.02, respectively, compared with the control (5.74 ± 0.021, *p*-value = 0.01).

#### 3.5.4. Crust and Crumb Color

Crust Color: The incorporation of chamomile powder resulted in significantly darker crusts compared with those of the control. The L* value decreased from 61.15 ± 2.47 in the control to 44.9 ± 4.67 for BI-MC and 11.55 ± 0.35 for BP-MC, indicating a darker color due to Maillard reactions and caramelization during baking. The a* value for BP-MC (5.85 ± 0.45) was significantly higher than that of the control (−0.75 ± 2.19), indicating more redness in the crust.

Crumb Color: The L* value of the crumb for BP-MC was significantly lower (17.7 ± 2.3) compared with those of BI-MC (37.55 ± 5.02) and the control (31.95 ± 0.49). The a* value for BP-MC was significantly higher (7.3 ± 0.4) than that of the control (4 ± 0.28), suggesting that BP-MC resulted in a redder crumb, likely due to the flavonoid content in chamomile.

#### 3.5.5. Crumb Structure

BI-MC 30% exhibited a significantly higher number of cells per square millimeter (220.11 ± 41.01) compared with BP-MC 30% (104.03 ± 7.07). However, BP-MC 30% had significantly larger cells and a longer cell perimeter (129.73 ± 6.54) compared to BI-MC 30%.

### 3.6. Sensory Evaluation of Enriched Breads

**Figure 2:**
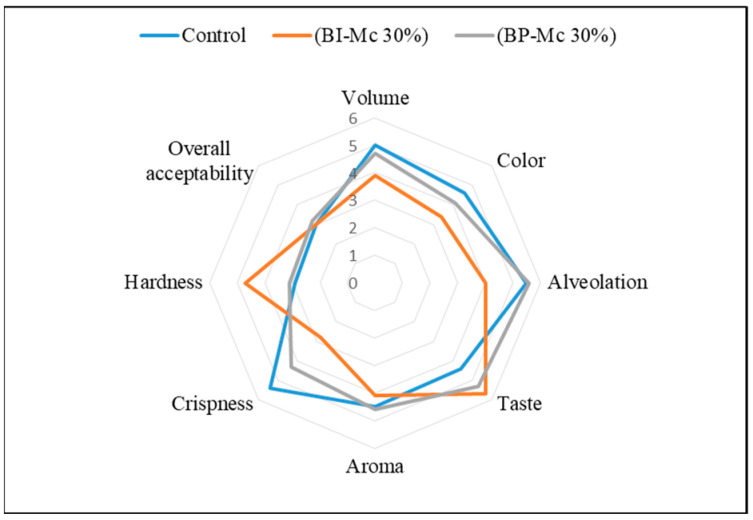
Sensory quality traits of bread samples enriched with 30% *Matricaria chamomilla* in powder and infusion forms. The sensory attributes evaluated include color, taste, texture, aroma, crispness, and overall acceptability.

Sensory analysis showed that the inclusion of 30% chamomile powder, both in powder (BP-MC 30%) and infusion (BI-MC 30%) forms, impacted the texture and crispness of the bread (Figure 2). Specifically, crispness was significantly lower in BI-MC 30% (2.80 ± 1.62) compared with the control bread (5.40 ± 1.43), with BP-MC 30% showing an intermediate value (4.30 ± 1.42). Additionally, the bread texture (hardness) was rated as more favorable in BP-MC 30% (3.10 ± 1.73) compared with the control (2.90 ± 0.57), whereas BI-MC 30% (4.70 ± 2.16) exhibited a higher hardness value, suggesting a firmer texture.

### 3.7. Total Phenolic and Flavonoid Content and Antioxidant Activity of Enriched Breads

**Figure 3:**
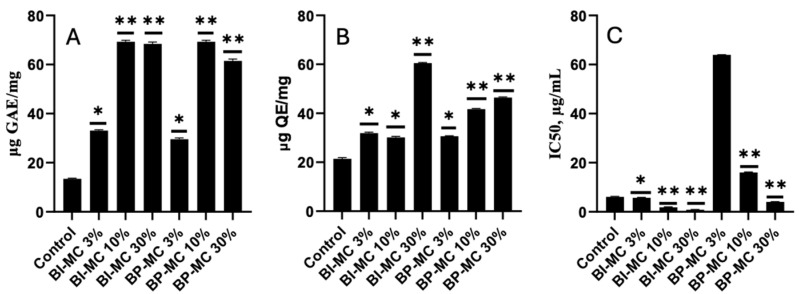
Total phenolic (**A**) and flavonoid contents (**B**), and antioxidant activity (**C**) of chamomile-enriched bread. The values represent means ± standard deviations from triplicate measurements. * Significance levels are indicated (* *p* < 0.05, ** *p* < 0.01) where applicable. GAE: Gallic Acid Equivalent, QE: Quercetin Equivalent; Vitamin C was used as a reference in the DPPH assay.

The incorporation of *Matricaria chamomilla* into bread significantly enhanced both the total phenolic and flavonoid contents compared with the control. The control bread exhibited 13.41 μg GAE/mg for phenolic content and 21.46 μg QE/mg for flavonoid content. Bread enriched with chamomile infusion (BI-MC) showed a substantial increase in both phenolic (68.41 μg GAE/mg) and flavonoid contents (30.18 μg QE/mg) at 10%, and the highest phenolic content (69.29 μg GAE/mg) and flavonoid content (60.47 μg QE/mg) at 30%. Statistical analysis (e.g., ANOVA, Tukey’s test) confirmed no significant difference between the 10% and 30% concentrations in terms of phenolic content (*p* > 0.05), though there was a significant difference between 3% and 30% for both phenolic and flavonoid contents (*p* < 0.05). On the other hand, the bread enriched with chamomile powder (BP-MC) exhibited promising antioxidant and polyphenol content, ranging from 29.59 μg GAE/mg at 3% to 61.48 μg GAE/mg at 10%, and 61.48 μg GAE/mg at 30%. The flavonoid content for BP-MC ranged from 30.63 μg QE/mg at 3% to 46.46 μg QE/mg at 30%. The antioxidant activity, measured through the DPPH assay, revealed that BI-MC-enriched breads had significantly higher antioxidant capacity than both the BP-MC-enriched and control breads. The IC50 value for BI-MC at a 30% concentration was 0.86 µg/mL, which was notably lower than that of the control (6.1 µg/mL) and BP-MC at 30% (4.05 µg/mL) (Figure 3 and Table S8).

### 3.8. Hierarchical Cluster Analysis of Bread Formulations

**Figure 4:**
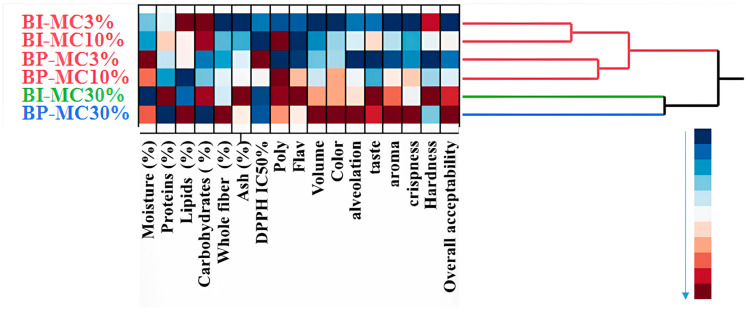
Heatmap of physicochemical, antioxidant, and sensory characteristics of six breads with different concentrations (3%, 10%, and 30%) of MC powder (BP-MC) and infusion (BI-MC).

A hierarchical cluster analysis (HCA) was performed to categorize the breads based on their physicochemical, antioxidant, and sensory properties. Figure 4 shows a heatmap of the physicochemical, antioxidant, and sensory characteristics of the six breads with different concentrations (3%, 10%, and 30%) of MC infusion and powder. This heatmap visually represents the concentration of key attributes, such as protein, fiber, antioxidants (polyphenols and flavonoids), and sensory attributes (volume, color, taste, crispness). The grouping of bread samples based on these attributes is clearly demonstrated. The analysis revealed three distinct clusters based on similarities in composition and sensory characteristics.

Cluster 1: BI-MC 3%, BI-MC 10%, BP-MC 3%, BP-MC 10%;

Cluster 2: BI-MC 30%;

Cluster 3: BP-MC 30%.

Breads in Cluster 1 (BI-MC 3%, BI-MC 10%, BP-MC 3%, and BP-MC 10%) exhibited similar characteristics, including lower fiber, ash, polyphenol, and flavonoid contents, as well as lower sensory quality scores. Specifically, the bread samples in this cluster had the lowest levels of total polyphenols (e.g., 33.12 Œºg QE/mg for BI-MC 3%) and flavonoids (e.g., 31.88 Œºg EQ/mg for BP-MC 3%) compared with the higher concentration samples. These breads were also rated lower in sensory attributes, particularly in terms of taste and texture, with sensory scores for aroma and crispness being modest (e.g., 2.70 for taste in BI-MC 3%).

In contrast, Cluster 2 (BI-MC 30%) showed higher protein, carbohydrate, and ash contents, along with elevated polyphenol and flavonoid levels. For example, the bread enriched with 30% chamomile infusion (BI-MC 30%) demonstrated a significant increase in polyphenol content (68.41 Œºg QE/mg) and flavonoids (60.47 Œºg EQ/mg) compared with Cluster 1 breads. Sensory evaluation for BI-MC 30% also indicated higher ratings for taste (5.70), hardness (4.70), and overall acceptability (3.00), making it a preferable choice in terms of both composition and sensory qualities.

**Figure 5:**
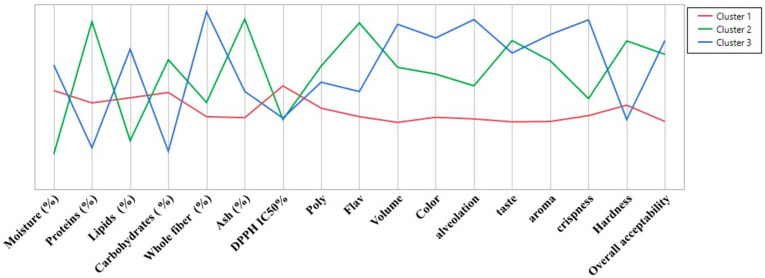
Clustering of bread samples based on their physicochemical, antioxidant, and sensory characteristics.

Finally, Cluster 4 (BP-MC 30%) demonstrated the highest levels of water, lipids, and fiber, alongside superior sensory attributes, such as enhanced volume (4.7), color (4.1), alveolation (5.6), and crispness (4.3). The total polyphenol (61.48 Œºg QE/mg) and flavonoid (41.67 Œºg EQ/mg) contents in BP-MC 30% were significantly higher than those in the other samples in Cluster 1, which contributed to its enhanced sensory quality. Breads in this cluster were rated highest for overall acceptability (3.2) and exhibited the most desirable qualities in terms of appearance and texture, highlighting the superior effect of 30% chamomile powder in enhancing both physicochemical and sensory attributes. A hierarchical clustering analysis of bread samples based on their physicochemical, antioxidant, and sensory characteristics is presented in Figure 4 and Figure 5. This clustering analysis groups the six bread samples into three distinct clusters, with the higher concentrations of chamomile infusion (BI-MC 30%) and powder (BP-MC 30%) showing a marked improvement in bread quality compared with lower concentrations (3% and 10%).

## 4. Discussion

The results of this study provide new insights into the potential of chamomile (*Matricaria chamomilla*) as a functional ingredient in bread, both in powdered and infusion forms. The incorporation of chamomile into bread led to significant improvements in nutritional composition, antioxidant activity, and sensory characteristics [34]. These findings not only confirm the initial hypotheses regarding the functionality of chamomile in enhancing bread quality, but also suggest potentially useful applications in improving the nutritional value of baked goods and in the sustainable use of plant by-products, traditionally underutilized.

### 4.1. Potential Functional and Nutritional Benefits of Chamomile’s Aerial Parts

The distinct phytochemical profiles of the *Matricaria chamomilla* infusion and ethanolic extract reflect the influence of the extraction method on the retention of bioactive compounds. The infusion, characterized by hydrophilic phenolic acids like caffeoylquinic acid derivatives, is particularly rich in antioxidants due to the water solubility of these compounds. These compounds are often associated with various health benefits, including antioxidant and anti-inflammatory effects [35]. On the other hand, the ethanolic extract, which contains higher concentrations of flavonoid glycosides such as luteolin O-glucoside and apigenin O-diglucuronide, is better suited for applications requiring higher polyphenol concentrations [36,37]. These flavonoids are known for their antioxidant and anti-inflammatory properties, and the ethanolic extraction method proves more effective in isolating these compounds, which are less soluble in water. The analysis of chamomile-enriched bread, particularly bread enriched with the ethanolic extract (BE-MC), confirmed that the baking process preserved these polyphenolic compounds. This demonstrates that the ethanolic extract can be effectively utilized in bakery products, enhancing their nutritional profile without compromising the bioactive properties. The UHPLC-HRMS method developed in this study proves to be a reliable tool for monitoring and evaluating the retention of bioactive compounds in chamomile-enriched foods. Overall, these findings underline the potential of chamomile as a functional ingredient in bakery products, with both the infusion and ethanolic extract offering distinct bioactive properties that can be harnessed for health-promoting foods.

In terms of antioxidant activity, the results from the DPPH, ABTS, and CUPRAC assays revealed that the ethanol extract exhibited higher antioxidant capacity than the chamomile infusion. These findings are consistent with those of previous studies, which have reported that ethanol extraction tends to yield higher antioxidant activity due to the increased solubility of phenolic compounds, especially flavonoids [37]. For instance, studies have highlighted that ethanol extracts of chamomile are particularly rich in flavonoids, which are well-known for their potent antioxidant properties [36,38]. However, while the ethanol extract exhibited higher antioxidant activity in these assays, the chamomile infusion, despite being slightly less potent, still represents a viable option for functional applications in food. The chamomile infusion offers notable advantages in terms of ease of preparation and cost-effectiveness, making it a sustainable and accessible option for functional food applications, especially in industrial settings like bakery products. Water-based extracts align with the growing demand for sustainable food production, as they are easy to incorporate into products while maintaining consumer appeal. Additionally, these extracts support the recovery of bioactive compounds from food waste through eco-friendly methods, promoting green chemistry and sustainability [39].

Both the chamomile infusion and ethanolic extract also demonstrated significant α-amylase inhibitory activity. This is in agreement with findings in the literature that flavonoids, such as apigenin and luteolin, found in chamomile can inhibit α-amylase activity, potentially leading to reduced starch digestion and improved glycemic control [40,41]. The administration of ethanolic chamomile extract in diabetic rats resulted in a decrease in postprandial hyperglycemia, decreased oxidative stress, and increased antioxidant activity, reinforcing chamomile’s potential as a therapeutic agent for metabolic diseases [42]. Chamomile water extracts, such as chamomile tea, have also shown moderate suppression of hyperglycemia in both sucrose-loading and streptozotocin (STZ)-induced diabetic rat models [43]. Additionally, recent reviews have emphasized chamomile’s broader potential in managing metabolic disorders, such as diabetes and obesity, by not only modulating enzymatic activity, but also influencing oxidative stress and metabolic profiles [44,45]. These findings suggest that chamomile supplementation, whether as ethanolic extract, aqueous extract, or tea, can positively impact blood glucose levels, insulin sensitivity, and oxidative stress. For example, together, these results suggest that chamomile not only inhibits α-amylase activity, but also offers broader metabolic benefits, such as improving glycemic control and reducing oxidative damage. The α-amylase inhibitory activity described above is particularly relevant in the context of food production, especially in products like bread. During baking, starches in flour undergo gelatinization, and their digestion plays a pivotal role in the postprandial glycemic response. By incorporating chamomile extracts into bread, it may be possible to modulate starch digestion, potentially resulting in a product with a lower glycemic index. Studies have highlighted the critical role of food structure in modulating starch digestibility and glycemic responses [46,47,48]. Research on bread structure has shown that variations in crumb texture can significantly alter the rate at which starch is digested, suggesting that modifying food matrix microstructure can control the release of glucose [49]. Breads with different crumb structures demonstrated notable differences in digestion, reinforcing the idea that altering the physical structure of starch can impact its bioavailability. Polyphenols, such as those found in chamomile, can either inhibit digestive enzymes directly (e.g., α-amylase) or form inclusion complexes with starch, thereby reducing its digestibility [50,51]. This process is particularly important when considering the dietary management of conditions like type II diabetes, where controlling postprandial glucose spikes is critical. For instance, chamomile’s aqueous extract, rich in flavonoids, reduced α-glucosidase activity by 60% at a concentration of 0.5 mg/mL [40], highlighting its role as a potent starch digestion inhibitor. In addition to enzyme inhibition, polyphenols can form non-covalent complexes with starch, which further limits the enzyme’s ability to hydrolyze starch. These interactions are typically driven by hydrogen bonds and hydrophobic interactions, reducing the accessibility of starch to digestive enzymes. For example, when phenolic compounds are incubated with the starch matrix, they can form complexes that reduce enzyme accessibility, further slowing the digestion process [51]. Our study suggests that chamomile extracts, rich in polyphenols, may provide a dual benefit: modifying the bread’s microstructure to limit enzyme access to starch and directly inhibiting α-amylase activity, which could slow down starch digestion and lower the glycemic index. This strategy could be especially beneficial for individuals with diabetes or those aiming to manage blood glucose levels.

### 4.2. Effect of Chamomile Incorporation in Bread

The incorporation of chamomile into bread formulations resulted in significant changes to its nutritional composition, notably in terms of protein, fiber, and micronutrient content. These alterations can enhance the functional properties of the bread, making it a potential vehicle for the delivery of bioactive compounds, which is of increasing interest within the food industry and for public health. The protein content of BI-MC (bread enriched with chamomile infusion) was significantly higher (14.78%) than those of both the control bread (12.07%) and BP-MC (bread enriched with chamomile powder), which had a protein content of 12.06%. This finding may be explained by the higher bioavailability of water-soluble proteins in the BI-MC formulation. The aqueous extraction of proteins is a commonly used extraction method for various protein sources [52]. In terms of dietary fiber, BP-MC (bread enriched with chamomile powder) exhibited the highest increase, reaching 10.6%, which was an improvement over 20% compared with the control bread (2.7%). The fiber content in BI-MC was also significantly higher (3.93%) compared with that of the control, though lower than that of BP-MC. This difference in fiber content between the two forms of chamomile highlights the potential benefits of using powdered chamomile as a source of dietary fiber in bakery products. Chamomile powder (CR) contains significant quantities of fiber (18.80 g/100 g) and protein (14.80 g/100 g), along with higher concentrations of sulfur amino acids [53]. Its incorporation into bread provides an effective strategy for boosting fiber intake, which is often inadequate in many populations [54]. Increasing fiber intake has been associated with numerous health benefits, including improved digestive health, weight management, and reduced risk of chronic diseases, such as cardiovascular diseases and type 2 diabetes [55]. The increased fiber content in BP-MC can be particularly advantageous in promoting gut health and regulating blood sugar levels, as dietary fiber plays a key role in these processes [56,57]. Another important consideration is the moisture content of the enriched breads. Both BI-MC and BP-MC breads showed lower moisture content compared with that of the control, with BI-MC exhibiting the lowest moisture level (12.55%). BP-MC had a higher moisture content (30.74%), though still significantly lower than that of the control bread (40.67%). The reduction in moisture content in both chamomile-enriched breads can likely be attributed to the hygroscopic properties of fiber, which binds water in the dough [58]. A lower moisture content may result in a firmer and denser texture, which could influence the bread’s shelf life and freshness [59]. While some consumers may prefer a firmer texture, this change could pose challenges in terms of sensory attributes, such as softness and overall acceptability. This reduction in moisture may also limit the product’s shelf-life, as lower moisture levels typically reduce microbial growth, but can also affect the freshness and palatability of the bread. To mitigate this issue, future studies could explore methods to enhance the texture and moisture retention of the enriched bread, perhaps through the addition of natural humectants or other ingredients that preserve moisture [60]. Furthermore, both BI-MC and BP-MC breads showed higher ash content (8.48% for BI-MC and 6.17% for BP-MC) compared with the control bread (4.33%). This increase in ash content indicates a higher mineral content, which is consistent with the known presence of various minerals in chamomile, such as calcium, magnesium, and potassium [61]. Minerals are essential for various physiological functions, including bone health, muscle function, and electrolyte balance. Therefore, the increased ash content of the enriched breads suggests that chamomile could be a beneficial source of bioavailable minerals, which could contribute to improved nutritional intake. Lipids were also affected by the incorporation of chamomile, with BI-MC having the lowest lipid content (0.8%), followed by BP-MC (1.2%) and the control bread (1.8%). This reduction in lipid content is likely due to the dilution effect of the chamomile and the lower fat content of the herb itself. While the decrease in lipid content is not necessarily problematic, it may impact the sensory profile of the bread, particularly in terms of its mouthfeel and richness. Lipids are crucial for the palatability of baked goods, as they contribute to the bread’s softness and moistness. Therefore, the reduction in fat may affect consumer acceptance, and future research could investigate ways to balance the nutritional benefits of reduced lipids with the sensory characteristics desired in bakery products. These findings suggest that chamomile-enriched breads could serve as a valuable functional food, addressing the growing demand for plant-based and health-promoting bakery products. The differences in nutritional composition between BI-MC and BP-MC breads highlight the distinct advantages of using chamomile in different forms, depending on the desired nutritional outcomes. The BI-MC bread, with its higher protein and lower lipid content, could appeal to consumers seeking plant-based protein, while BP-MC bread, with its elevated fiber content, may be particularly beneficial for individuals looking to increase their dietary fiber intake. The incorporation of *Matricaria chamomilla* into bread significantly influenced the bioactive compound profile, particularly in terms of phenolic compounds and antioxidant activity, as reflected in the results presented in Figure 3. The antioxidant activity, evaluated through the DPPH assay, followed a similar trend. The IC50 values demonstrated that BI-MC-enriched breads exhibited significantly stronger antioxidant activity than the BP-MC-enriched or control breads. At 30% infusion concentration, the IC50 value reached 0.86 µg/mL, which was substantially lower than that of the control (6.1 µg/mL), indicating a higher antioxidant capacity. The antioxidant activity in BP-MC-enriched breads was also active, with IC50 values ranging from 4.05 µg/mL at 30%, which is better than that of the control. These findings underline the significant role of chamomile as a functional ingredient in bread, particularly in enhancing its antioxidant profile. This could offer a functional food product that not only improves the nutritional profile, but also provides antioxidant protection, which is beneficial for reducing oxidative stress and mitigating the risk of chronic diseases, such as cardiovascular conditions and cancer [62].

The sensory analysis revealed that chamomile infusion (30%) in bread created a light herbal aroma, which was generally well-received by the panelists. This pleasant, mild herbal note is consistent with findings [53] reporting that herbs impart delicate aromas to baked goods. On the other hand, bread enriched with chamomile powder, though richer in flavor, had a slightly bitter aftertaste, which some panelists found less appealing. This bitterness could be attributed to the higher concentration of bioactive compounds like flavonoids present in the powder, which are known to contribute to a bitter flavor profile [63]. The results suggest that, while the chamomile infusion offers a more subtle, consumer-friendly aroma, the stronger, potentially bitter flavor of the chamomile powder may limit consumer acceptance, particularly among those unaccustomed to herbal flavors in bread. Additionally, the texture evaluation indicated an increase in bread firmness with the addition of chamomile, particularly at higher concentrations. This increase in firmness is likely due to the fiber content in chamomile and, hence, the presence of hydrocolloids, which act as thickening, texturizing, stabilizing, and gelling agents [64]. The denser, firmer texture of chamomile-enriched bread may appeal to consumers seeking a heartier product, but it may be a disadvantage for those who prefer softer, airier breads. These findings suggest a trade-off between the nutritional benefits of chamomile (e.g., higher fiber content) and the texture preferences of consumers. The results of the hierarchical cluster analysis (HCA) further support the sensory observations. The analysis grouped the bread samples into three distinct clusters based on physicochemical, antioxidant, and sensory properties (Figure 4 and Figure 5). Cluster 1 contained breads with lower concentrations of chamomile (3% and 10%) and exhibited modest sensory ratings, particularly in terms of taste and texture. These breads, which had lower levels of polyphenols and flavonoids, were rated lower in sensory attributes, such as aroma and crispness, with a taste score of 2.5 for BI-MC 3%. In contrast, Cluster 2 (BI-MC 30%), showed the highest levels of polyphenols, flavonoids, and antioxidant capacity, with sensory scores also showing some positive trends, particularly for taste. However, sensory evaluation revealed no significant differences in overall acceptability between BI-MC 30%, BP-MC 30%, and the control bread. Cluster 3 (BP-MC 30%), enriched with 30% chamomile powder, demonstrated significant improvements in polyphenol and flavonoid content. While sensory evaluation revealed some differences in individual attributes (such as taste and texture), the overall acceptability of BI-MC 30%, BP-MC 30%, and the control bread did not show significant differences, highlighting that the addition of chamomile in both forms did not notably affect the consumers’ overall perception of the bread

## 5. Conclusions

This study confirmed that fortifying bread with 30% *Matricaria chamomilla* (in powder or infusion form) significantly enhances its nutritional value while maintaining sensory attributes similar to those of the control bread. The addition of chamomile increased the antioxidant activity, particularly due to the presence of polyphenols, improving the nutritional profile without drastically altering the bread’s texture and flavor. This is desirable for consumers, as it offers health benefits while preserving familiar sensory characteristics. Specifically, chamomile powder boosted the protein content by 15% and total fiber by 20%, positioning it as a valuable ingredient for enhancing the nutritional composition of bread. Sensory evaluations showed that, while the chamomile infusion provided a subtle herbal aroma and was well received, the chamomile powder imparted a slightly bitter taste, which may limit its appeal to some consumers. Additionally, the increased fiber content led to a firmer texture, which might be preferred by some, but could be less favorable for others who enjoy softer bread. These findings highlight the potential of chamomile as a functional ingredient that adds nutritional value while keeping sensory properties close to those of traditional bread. Further studies are needed to optimize the formulation, balancing the nutritional advantages with sensory qualities, such as taste and texture. Future research could also explore different chamomile concentrations, processing methods, and storage conditions to improve stability and sensory characteristics. By addressing these challenges, chamomile-enriched bread could become a valuable addition to the growing market for functional foods, combining tradition with innovation.

## Figures and Tables

**Table 1:** UHPLC-(-)HRMS data of compounds detected in *Matricaria chamomilla* ethanolic extract (MC-E), infusion (MC-I), and bread fortified with ethanolic extract (BE-MC).

ID	Name	Molecular Formula	RT (min)	Precursor [M-H]^−^	NEG ppm	Confirming Fragment	MSI Level	Samples
MC_1	Caffeoyl quinic acid	C_16_H_18_O_9_	5.28	353.0886	2.04	191.0553, 179.0341, 135.0440	2	MC-E, MC-I, BE-MC
MC_2	Caffeoyl hexoside	C_15_H_18_O_9_	5.55	341.0877	1.24	179.0341, 161.0246, 135.0443	2	MC-E, MC-I, BE-MC
MC_3	Glucosyringic acid	C_15_H_20_O_10_	5.75	359.0982	1.16	197.0457	2	MC-E, MC-I
MC_4	Caffeoyl quinic hexoside	C_22_H_28_O_14_	5.76	515.1411	0.97	323.0777, 353.0882, 191.0553	2	MC-E, MC-I, BE-MC
MC_5	Eucomic acid	C_11_H_12_O_6_	6.02	239.0561	0.12	177.0559, 133.0661, 149.0609	2	MC-I
MC_6	Caffeoyl hexoside	C_15_H_18_O_9_	6.23	341.0877	1.24	179.0341, 135.0443	2	MC-E, MC-I, BE-MC
MC_7	Coumaroylquinic acid	C_16_H_18_O_8_	6.55	337.0931	0.53	163.0403, 191.0550	2	MC-I
MC_8	Esculetin	C_22_H_28_O_14_	6.99	177.0194	0.33	133.0297, 105.0347	2	MC-E, MC-I, BE-MC
MC_9	Dihydroxybenzoic acid di-pentoside	C_17_H_22_O_12_	7.02	417.1036	−0.57	285.0617, 241.0718, 152.0117, 108.0218	2	MC-I
MC_10	Caffeoyl quinic hexoside	C_9_H_6_O_4_	7.18	515.1411	0.97	323.0777, 353.0882, 191.0553, 161.0246	2	MC-E, MC-I, BE-MC
MC_11	Caffeic acid	C_9_H_8_O_4_	7.3	179.035	0.04	135.0453	2	MC-E, MC-I, BE-MC
MC_12	Caffeoyl quinic acid	C_16_H_18_O_9_	7.47	353.0878	−0.25	191.0553	2	MC-E, MC-I, BE-MC
MC_13	Caffeoyl quinic acid	C_16_H_18_O_9_	7.58	353.0877	−0.31	191.0553, 173.0457, 135.0454	2	MC-E, MC-I, BE-MC
MC_14	Caffeic acid derivative	C_17_H_18_O_10_	8.39	381.0825	−0.52	251.0562, 179.0341, 161.0234	3	MC-E, MC-I, BE-MC
MC_15	Feruloyl hexoside	C_16_H_20_O_9_	8.43	355.1034	−0.18	193.0508, 149.0610	2	MC-I
MC_16	Vanillic acid	C_8_H_8_O_4_	8.6	167.035	0.05	152.0117, 108.0218	2	MC-E, MC-I, BE-MC
MC_17	Tuberonic acid	C_18_H_28_O_9_	8.91	387.1657	−0.35	207.1028	2	MC-E, MC-I, BE-MC
MC_18	Coumaroylquinic acid	C_16_H_18_O_8_	9.55	337.0931	0.53	163.0403, 191.0550	2	MC-E, MC-I, BE-MC
MC_19	Hydroxyjasmonic acid	C_12_H_18_O_4_	9.63	225.1133	0.21	97.0259, 59.0139	2	MC-I
MC_20	Verbasoside	C_20_H_30_O_12_	10.59	461.1667	−0.48	191.0563, 149.0457, 131.0351	2	MC-E, MC-I
MC_21	Feruloylquinic acid	C_17_H_20_O_9_	11.52	367.1035	−0.5	191.0563, 173.0457	2	MC-E, MC-I, BE-MC
MC_22	Apigenin-di C-hexoside	C_17_H_30_O_15_	12.16	593.1517	1.72	473.1095, 383.0772, 353.0666	2	MC-E, MC-I, BE-MC
MC_23	Myricetin O-hexoside	C_21_H_20_O_13_	13.48	479.0829	−0.83	317.0301	2	MC-E, BE-MC
MC_24	Apigenin O-diglucuronide	C_27_H_26_O_17_	14.02	621.1094	−0.26	351.0567, 269.0455	2	MC-I
MC_25	Quercetin O-hexoside	C_21_H_20_O_12_	14.25	463.0889	0.86	301.0353	2	MC-E, BE-MC
MC_26	Luteolin O-glucoside	C_21_H_20_O_11_	14.34	447.0931	−0.82	285.0402, 151.0038, 133.0293	2	MC-E, MC-I, BE-MC
MC_27	Patuletin O-hexoside	C_22_H_22_O_13_	14.38	493.0991	0.25	331.0459, 316.0220	2	MC-E, MC-I, BE-MC
MC_28	Dicaffeoylquinic acid	C_25_H_24_O_12_	14.62	515.1191	−0.67	353.0878, 191.0553, 135.0439	2	MC-E, MC-I, BE-MC
MC_29	Methylquercetin O-gluronide	C_22_H_20_O_13_	14.68	491.0837	0.46	315.0506, 300.0274	2	MC-E, MC-I, BE-MC
MC_30	Dicaffeoylquinic acid	C_25_H_24_O_12_	14.78	515.1191	−0.67	353.0878, 191.0553, 135.0439	2	MC-E, MC-I, BE-MC
MC_31	Apigenin O-gluronide	C_21_H_18_O_11_	14.51	445.0774	−1.21	269.0455	2	MC-E, MC-I, BE-MC
MC_32	Apigenin O-hexoside	C_21_H_20_O_10_	14.85	431.0979	−1.73	268.0378	2	MC-E, MC-I, BE-MC
MC_33	Caffeic acid derivative	C_26_H_24_O_13_	14.91	543.1141	−0.83	381.0821, 161.0233	3	MC-E, MC-I, BE-MC
MC_34	Dicaffeoylquinic acid	C_25_H_24_O_12_	15.07	515.1191	−0.67	353.0878, 191.0553, 135.0439	2	MC-E, MC-I, BE-MC
MC_35	Luteolin O-caffeoylhexoside	C_30_H_26_O_14_	15.46	609.1261	1.64	285.0403, 161.0233	2	MC-E, MC-I, BE-MC
MC_36	Apigenin O-caffeoylhexoside	C_30_H_26_O_13_	15.86	593.1307	0.9	323.0771, 269.0455, 161.0234	2	MC-E, MC-I, BE-MC
MC_37	Luteolin	C_15_H_10_O_6_	15.95	285.0406	0.14	151.0038, 133.0296	2	MC-E, MC-I, BE-MC
MC_38	Methylquercetin	C_16_H_12_O_7_	16.00	315.0515	0.97	300.0274	2	MC-E, MC-I, BE-MC
MC_39	Caffeic acid derivative	C_35_H_30_O_16_	16.03	705.1466	−1.25	543.1141, 381.0821, 161.0233	3	MC-E, MC-I, BE-MC
MC_40	Trihydroxyoctadecadienoic acid	C_18_H_32_O_5_	16.20	327.2174	−0.88	229.1439, 211.133	2	MC-E, MC-I, BE-MC
MC_41	Apigenin	C_15_H_10_O_5_	16.27	269.0453	−1.45	227.0352, 151.0026, 117.0347	2	MC-E, MC-I, BE-MC
MC_42	Hispidulin	C_16_H_12_O_6_	16.30	299.0559	−0.84	284.0325	2	MC-E, MC-I, BE-MC
MC_43	Trihydroxyoctadecenoic acid	C_18_H_34_O_5_	16.32	329.2335	0.34	229.1439, 211.133	2	MC-E, MC-I, BE-MC

**Table 2:** Total phenolic and flavonoid contents, antioxidant, and antidiabetic activity of *Matricaria chamomilla* infusion (Mc-I) and ethanolic extract (Mc-EtOH).

Extracts/Standards	Total Phenolic Content (µg GAE/mg)	Total Flavonoid Content (µg QE/mg)	Antioxidant Activity (IC50 µg/mL)	Antidiabetic Activity (IC50 µg/mL)
	DPPH Assay	ABTS Assay	CUPRAC Assay	Acarbose (A50 µg/mL)
Mc-EtOH	110.32 ± 0.62	89.79 ± 0.58	13.15 ± 0.95	81.04 ± 0.42
Mc-	82.82 ± 0.41	47.91 ± 0.29	24.46 ± 0.35	93.91 ± 0.94
BHT	NT	NT	12.99 ± 0.41	1.29 ± 0.30
BHA	NT	NT	6.14 ± 0.41	1.81 ± 0.10
Acarbose	NT	NT	NT	NT

NT = Not tested: The values represent means ± standard deviations from triplicate measurements.

**Table 3:** Nutritional composition of control bread and bread enriched with two different *Matricaria chamomilla* extracts: BI-Mc 30% (infusion-based extract) and BP-Mc 30% (ethanolic extract).

Bread Traits (%)	Control	Bread (BI-Mc 30%)	Bread (BP-Mc 30%)	*p*-Value	Significance
Moisture	40.67 ± 0.58 ^a^	12.55 ± 0.69 ^c^	30.74 ± 0.81 ^b^	0.0001	***
Proteins	12.07 ± 0.92 ^b^	14.78 ± 0.66 ^a^	12.06 ± 0.17 ^b^	0.003	**
Lipids	1.80 ± 0.01 ^a^	0.80 ± 0.10 ^c^	1.20 ± 0.20 ^b^	0.0002	***
Carbohydrates	38.44 ± 0.57 ^b^	59.46 ± 0.61 ^a^	39.23 ± 0.80 ^b^	0.0001	***
Whole Fiber	2.70 ± 0.50 ^b^	3.93 ± 0.53 ^b^	10.6 ± 0.54 ^a^	0.0001	***
Ash	4.33 ± 0.58 ^c^	8.48 ± 0.75 ^a^	6.17 ± 0.76 ^b^	0.001	**

Values are presented as mean ± standard deviation (SD). Different letters within the same row indicate significant differences (*p* < 0.05). *p*-value: Values below 0.05 indicate statistical significance. Significance: *** *p* < 0.001, ** *p* < 0.01.

**Table 4:** Bread traits and sensory evaluation of chamomile-enriched reads.

Bread Traits	Control	Bread (BI-MC 30%)	Bread (BP-MC 30%)	*p*-Value	Significance
Vsp (cm^3^/g)	3.36 ± 0.001 ^a^	2.19 ± 0.07 ^b^	2.26 ± 0.20 ^b^	0.004	**
Weight loss (%)	31.30 ± 0.95 ^a^	30.04 ± 0.84 ^a^	32.69 ± 1.32 ^a^	0.18	ns
pH value	5.74 ± 0.02 ^a^	5.30 ± 0.02 ^b^	5.43 ± 0.02 ^b^	0.01	*
Crust color					
L*	61.15 ± 2.47 ^a^	44.9 ± 4.67 ^b^	11.55 ± 0.35 ^c^	0.001	**
a*	−0.75 ± 2.19 ^b^	4.00 ± 0.99 ^ab^	5.85 ± 0.45 ^a^	0.04	*
b*	26.90 ± 3.68 ^a^	30.65 ± 0.92 ^a^	7.25 ± 0.55 ^b^	0.003	**
Crumb color					
L*	31.95 ± 0.49 ^ab^	37.55 ± 5.02 ^ab^	17.70 ± 2.30 ^b^	0.02	*
a*	4.00 ± 0.28 ^ab^	−0.65 ± 2.76 ^b^	7.30 ± 0.40 ^a^	0.03	*
b*	24.10 ± 0.99 ^a^	27.05 ± 0.92 ^a^	16.80 ± 0.50 ^b^	0.002	**
Crumb structure					
Number of cells/mm^2^	153.00 ± 18.38 ^ab^	220.11 ± 41.01 ^a^	104.03 ± 7.07 ^b^	0.04	*
Average cell size (mm^2^)	500.96 ± 70.71 ^a^	485.86 ± 152.28 ^a^	1162.06 ± 243.03 ^a^	0.04	*
Area fraction (%)	35.51 ± 7.15 ^a^	50.74 ± 3.47 ^a^	49.87 ± 4.98 ^a^	0.11	ns
Perimeter	75.01 ± 5.28 ^b^	74.50 ± 5.61 ^b^	129.73 ± 6.54 ^a^	0.003	**
Circularity	0.75 ± 0.04 ^a^	0.76 ± 0.02 ^a^	0.77 ± 0.01 ^a^	0.77	ns
Solidity	0.85 ± 0.01 ^a^	0.84 ± 0.00 ^a^	0.85 ± 0.00 ^a^	0.52	ns
Sensory analysis attributes					
Volume	5.00 ± 0.94 ^a^	3.90 ± 1.85 ^a^	4.70 ± 2.16 ^a^	0.35	ns
Color	4.61 ± 1.51 ^a^	3.40 ± 1.90 ^a^	4.10 ± 2.38 ^a^	0.40	ns
Alveolation	5.51 ± 1.18 ^a^	4.00 ± 2.05 ^a^	5.61 ± 1.71 ^a^	0.07	ns
Taste	4.40 ± 1.07 ^a^	5.70 ± 1.16 ^a^	5.30 ± 2.21 ^a^	0.18	ns
Aroma	4.51 ± 1.08 ^a^	4.11 ± 1.29 ^a^	4.60 ± 2.12 ^a^	0.75	ns
Crispness	5.40 ± 1.43 ^a^	2.80 ± 1.62 ^b^	4.30 ± 1.42 ^ab^	0.002	**
Hardness	2.90 ± 0.57 ^a^	4.70 ± 2.16 ^a^	3.10 ± 1.73 ^a^	0.03	*
Overall acceptability	3.00 ± 0.82 ^a^	3.00 ± 0.67 ^a^	3.20 ± 0.79 ^a^	0.79	ns

Data are presented as mean ± standard deviation (SD). Statistical significance was determined using ANOVA followed by Tukey’s post hoc test. Significant differences between bread samples are indicated by different superscript letters (a, b, and c) within the same row (*p* < 0.05). Asterisks indicate the level of significance: * *p* < 0.05, ** *p* < 0.01.

## Data Availability

The original contributions presented in the study are included in the article, further inquiries can be directed to the corresponding author.

## Supplementary Materials

The following supporting information can be downloaded at: https://www.mdpi.com/article/10.3390/foods14050838/s1, Table S1: Antioxidant activity of *Matricaria chamomilla* infusion and ethanolic extract in DPPH assay; Table S2: Antioxidant activity of *Matricaria chamomilla* infusion and ethanolic extract in ABTS assay; Table S3: Absorbance of *Matricaria chamomilla* infusion and ethanolic extract in CUPRAC assay; Table S4: α-amylase inhibitory assay of *Matricaria chamomilla* infusion and ethanolic extract; Table S5: α-amylase inhibitory assay of acarbose used as standard; Table S6: Nutritional composition of wheat bread samples enriched with *Matricaria chamomilla* at 3%, 10%, and 30% concentrations; Table S7: Technological and sensory characteristics of wheat bread samples enriched with *Matricaria chamomilla* at 3%, 10%, and 30% concentrations; Table S8: Total phenolic, flavonoid contents, and antioxidant activity of bread enriched with *Matricaria chamomilla* at 3%, 10%, and 30% concentrations; Figure S1: Calibration curve of total phenolic content; Figure S2: Calibration curve of total flavonoid content; Figure S3: Bread during fermentation: Figure S4: Final bread product with 30% chamomile powder after baking; Figure S5: Sensory analysis sheet for bread evaluation; Figure S6: Inhibition curve of BP-Mc (30%) by DPPH assay; Figure S7: Inhibition curve of BI-Mc (30%) by DPPH assay.
