# Supplementary material for: Nutritional Composition, Physicochemical Properties, Antioxidant Activity, and Sensory Quality of Matricaria chamomilla-Enriched Wheat Bread
Source: Foods. 2025 Feb 28;14(5):838. doi: 10.3390/foods14050838 (PMC11898972; doi:10.3390/foods14050838)
Supplement: Supplementary file 1 [file foods-14-00838-s001.zip › foods-3475015-supplementary.pdf]

Supplementary Matherial

Nutritional Composition, Physicochemical Properties, Antioxidant Activity, and Sensory Quality of *Matricaria chamomilla*-Enriched Wheat Bread

Kerbab Khawla[1,2], Sanah Ibtissem[1,3], Djeghim Fairouze[4], Belattar Nadjah[2], Valentina Santoro[5,6], Maria D'Elia[5,6,7], Luca Rastrelli[5,6*]

[1]Laboratoire de Génie biologique Valorisation et innovation des Produits Agroalimentaires Institut ISTA- Ain
M'Lila, Université Larbi Ben M'hidi Oum El-Bouaghi, Algeria.
[2]Unité de recherche Valorisation des Ressources Naturelles, Molécules Bioactives et Analyses Physico
chimiques et Biologiques (VARENBIOMOL), Université Constantine 1,
Route de Aïn El Bey, Constantine, Algeria.
[3]Laboratoire de recherche en Sciences Alimentaires. Formulation. Innovation. Valorisation et Intelligence Art
ficielle (SAFIVIA). Institut de la Nutrition. de l'Alimentation et des
Technologies Agro-Alimentaires. (INATAA). Université frères Mentouri Constantine 1. Algeria
[4]Équipe FNPAA, Laboratoire de Nutrition et Technologie Alimentaire (L.N.T.A), Institut de la Nutrition, de
l'Alimentation et des Technologies Agro-Alimentaires (I.N.A.T.A. A), Université frères Mentouri Constantine
1. Algeria.
[5]Department of Pharmacy, University of Salerno, Via Giovanni Paolo II, 132, 84084 Fisciano, Italy;
[6]National Biodiversity Future Center—NBFC, 90133 Palermo, Italy
[7]Dipartimento di Scienze della Terra e del Mare, University of Palermo, Palermo, Italy

* *Correspondence to: Prof. Dr. Luca Rastrelli, Department of Pharmacy, University of Salerno, Via Giovanni Paolo II, Fisciano, 84084 Salerno, Italy; E-mail: rastrelli@unisa.it

TABLES S1-S8

Table S1. Antioxidant Activity of *Matricaria chamomilla* Infusion (Mc-I) and Ethanolic Extract (Mc-EtOH) in DPPH assay

Extract	% Inhibition in DPPH assay						
	12.5 µg/mL	25 µg/mL	50 µg/mL	100 µg/mL	200 µg/mL	400 µg/mL	800 µg/mL
Mc-EtOH	49.08±0.13	53.73±0.45	57.60±0.18	65.69±0.90	74.58±0.55	83.99±0.24	88.42±0.22
Mc-I	44.25±0.14	53.03±0.24	57.27±0.18	65.22±0.50	71.67±0.40	82.70±0.58	87.15±0.30
BHT	76.55±0.48	79.89±0.26	81.73±0.10	84.18±0.10	87.13±0.17	89.36±0.19	90.14±0.10
BHA	49.09±0.10	72.63±0.24	88.73±0.27	94.00±0.21	94.97±0.26	95.38±0.19	96.02±0.12

The values represent means ± standard deviations from triplicate measurements

Table S2. Antioxidant Activity of *Matricaria chamomilla* Infusion (Mc-I) and Ethanolic Extract (Mc-EtOH) in ABTS assay

Extract	% Inhibition in ABTS assay						
	12.5 µg/mL	25 µg/mL	50 µg/mL	100 µg/mL	200 µg/mL	400 µg/mL	800 µg/mL
Mc-EtOH	18.59 ± 0.57	26.54±0.75	40.68±1.08	56.44±0.59	64.52±0.56	76.17±0.89	78.87±0.66
Mc-I	20.62±1.04	25.89±0.79	37.23±0.85	51.43±0.56	66.66±0.97	73.81±1.41	79.88±0.39
BHT	69.83± 0.40	78.23±1.43	88.12± 0.90	88.76± 0.25	90.85± 1.74	90.95± 0.51	96.68± 0.39
BHA	92.83± 1.42	94.68±0.42	94.95± 0.90	95.32 ±0.25	95.59± 0.47	95.83± 0.15	95.8 ±0.10

The values represent means ± standard deviations from triplicate measurements

Table S3. Absorbance of *Matricaria chamomilla* Infusion (Mc-I) and Ethanolic Extract (Mc-EtOH) in CUPRAC assay

Extract	% Absorbance in CUPRAC assay						
	3.125 µg/mL	6.25 µg/mL	12.5 µg/mL	25 µg/mL	50 µg/mL	100 µg/mL	200 µg/mL
Mc-EtOH	0.33±0.01	0.62±0.03	0.83±0.08	1.38±0.15	1.94±0.10	2.51±0.17	3.02±0.07
Mc-I	0.37±0.01	0.65±0.02	0.93±0.04	1.45±0.10	2.06±0.02	2.70±0.07	3.29±0.20
BHA	1.41±0.03	2.22±0.05	2.42 ±0.02	2..50± 0.01	2.56± 0.05	2.86± 0.07	3.38±0.13
BHT	1.12± 0.05	1.95± 0.31	2.14±0.46	2.58± 0.42	3.35± 0.20	3.77± 0.19	3.92± 0.13

The values represent means ± standard deviations from triplicate measurements

Table S4. α-amylase inhibitory assay of *Matricaria chamomilla* Infusion (Mc-I) and Ethanolic Extract (Mc-EtOH)

Extract	1.9531 µg/mL	3.9062 µg/mL	7.812 µg/mL	15.625 µg/mL	31.25 µg/mL	62.5 µg/mL	125 µg/mL
Mc-EtOH	42.46±0.22	43.63±0.34	46.52±0.18	48.31±0.18	52.71±0.31	55.57±0.14	58.10±0.14
Mc-I	44.40±0.18	46.84±0.22	49.43±0.30	50.64±0.14	54.05±0.26	55.39±0.22	57.95±0.26

The values represent means ± standard deviations from triplicate measurements

Table S5. α-amylase inhibitory assay of Acarbose used as standard

Standard	62,5 µg	125 µg	250 µg	500 µg	1000 µg	2000 µg	4000 µg
Acarbose	7,76±0.1	8,08±0,3	9,46±0.1	10,70±0,6	31,81±2,9	37,21±3,4	53,05±1,9

The values represent means ± standard deviations from triplicate measurements

Table S6. Nutritional Composition of wheat bread samples enriched with *Matricaria chamomilla* at 3%, 10%, and 30% concentrations.

Sample	Moisture (%)	Proteins (%)	Lipids (%)	Carbohydrates (%)	Whole Fiber (%)	Ash (%)
Control	40.67 ± 0.58	12.07 ± 0.92	1.8 ± 0.02	38.44 ± 0.57	2.70 ± 0.50	4.33 ± 0.58
BI-MC (3%)	19.44 ± 0.71	13.02 ± 0.52	1.2 ± 0.11	60.40 ± 0.60	1.53 ± 0.20	4.33 ± 0.26
BI-MC (10%)	17.67 ± 0.43	13.54 ± 0.49	1.0 ± 0.04	59.56 ± 0.65	3.06 ± 0.40	5.17 ± 0.41
BI-MC (30%)	12.55 ± 0.69	14.78 ± 0.66	0.8 ± 0.10	59.46 ± 0.61	3.93 ± 0.53	8.48 ± 0.75
BP-MC (3%)	34.89 ± 0.91	12.93 ± 0.38	1.0 ± 0.04	42.547 ± 0.61	2.8 ± 0.44	5.83 ± 0.50
BP-MC (10%)	30.19 ± 0.79	12.53 ± 0.32	0.75 ± 0.03	46.367 ± 0.74	4.16 ± 0.51	6.00 ± 0.59
BP-MC (30%)	30.74 ± 0.81	12.06± 0.17	1.20 ± 0.20	39.23 ± 0.80	10.60 ± 0.54	6.17 ± 0.76

The values represent mean ± standard deviation; BI-MC: Bread enriched with *Matricaria chamomilla* infusion; BP-MC: Bread enriched with *Matricaria chamomilla* powder; Whole Fiber refers to the content of dietary fiber in the bread samples; The percentages in the table are based on the dry weight of the bread samples

Table S7. Technological and sensory characteristics of wheat bread samples enriched with Matricaria chamomilla at 3%, 10%, and 30% concentrations.

Sensory Attribute	BP-MC 3%	BP-MC 10%	BP-MC 30%	p-value	Significance	BI-MC 3%	BI-MC 10%	BI-MC 30%	p-value	Significance
Volume	3.0	3.2	4.7	0.11	NS	2.5	2.8	3.9	0.08	NS

Sensory Attribute	BP-MC 3%	BP-MC 10%	BP-MC 30%	p-value	Significance	BI-MC 3%	BI-MC 10%	BI-MC 30%	p-value	Significance
Color	2.6	3.4	4.1	0.19	NS	1.7	2.56	3.4	0.03	*
Alveolation	2.6	3.7	5.6	0.00	***	2.9	3.6	4.0	0.37	NS
Taste	2.3	3.1	5.3	0.00	***	2.7	4.2	5.7	0.00	***
Aroma	2.6	3.5	4.6	0.05	NS	2.5	3.2	4.1	0.02	*
Crispness	2.3	3.2	4.3	0.01	*	2.0	2.4	2.8	0.47	NS
Hardness	2.4	3.2	3.1	0.37	NS	4.5	3.5	4.7	0.35	NS
Overall acceptability	1.8	2.3	3.2	0.00	**	1.6	2.4	3.0	0.00	***

Significance: $p < 0.05$; **p** < 0.01; *__p__ < 0.001; NS = Not Significant

Table S8. Total phenolic, flavonoid contents, and antioxidant activity of bread enriched with Matricaria chamomilla at 3%, 10%, and 30% concentrations.

Bread Samples	Phenolic Content (µg GAE/mg)	Flavonoid Content (µg QE/mg)	DPPH Assay (IC50) µg/ml
**Control**	13.41 ± 0.10	21.46 ± 0.18	6.10 ± 0.24
**BI-MC 3%**	33.12 ± 0.23 *	31.88 ± 0.39 *	5.71 ± 0.16 *
**BI-MC 10%**	68.41 ± 0.75 **	30.18 ± 0.31 *	1.86 ± 0.07 **
**BI-MC 30%**	69.29 ± 0.76 **	60.47 ± 0.31 **	0.86 ± 0.13 **
**BP-MC 3%**	29.59 ± 0.17 *	30.63 ± 0.28 *	63.94 ± 0.59
**BP-MC 10%**	59.29 ± 0.80 **	41.67 ± 0.22 **	16.04 ± 0.36 **
**BP-MC 30%**	61.48 ± 0.76 **	46.46 ± 0.31 **	4.05 ± 0.14 **

Note: Phenolic content (µg GAE/mg), flavonoid content (µg QE/mg), and antioxidant activity (IC50 µg/ml) were measured in bread samples enriched with 3%, 10%, and 30% *Matricaria chamomilla* (BI-MC) and *Brewed Matricaria chamomilla* (BP-MC). Values are expressed as means ± standard deviation of three independent replicates. Statistical significance was determined using one-way ANOVA, followed by post-hoc analysis. Significant differences compared to the control are indicated by * ($p < 0.05$) and ** ($p < 0.01$).

FIGURES S1-S7

Figure S1. Calibration curve of Total Phenolic Content.

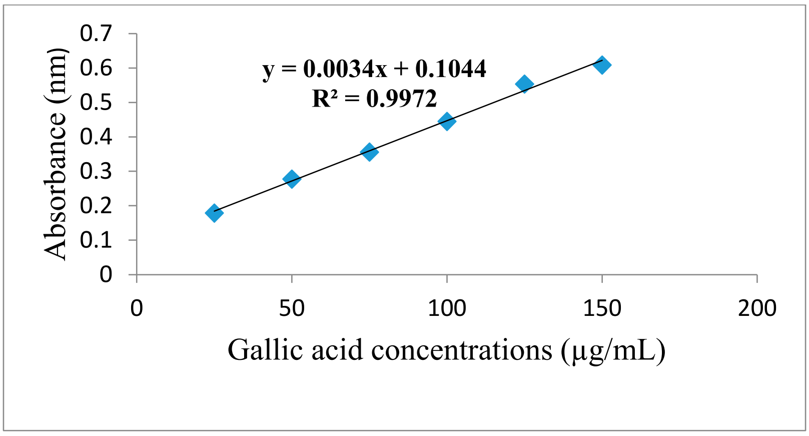

The calibration curve was constructed using a standard solution of gallic acid. The total phenolic content in the samples was determined based on this curve, and results are expressed as µg gallic acid equivalent (GAE) per gram of sample.

Figure S2. Calibration curve of Total Flavonoid Content.

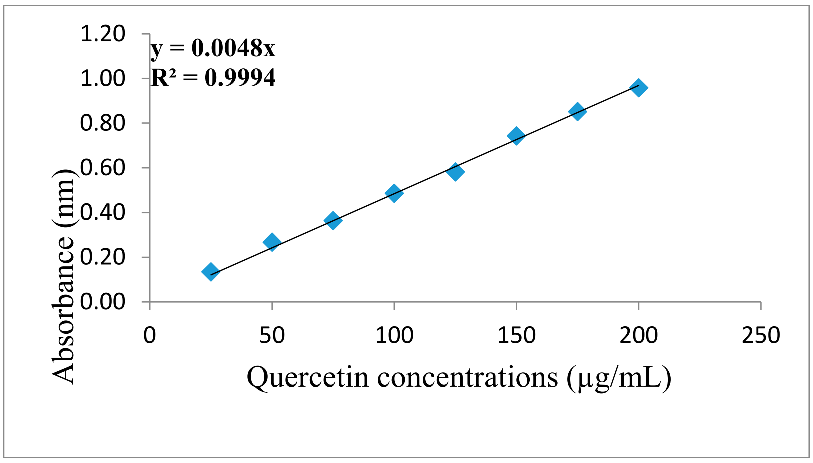

The calibration curve was constructed using a standard solution of Quercetin. The total flavonoid content in the samples was determined based on this curve, and results are expressed as µg quercetin equivalent (QE) per gram of sample.

Figure S3. Bread during fermentation, showing significant volume increase.

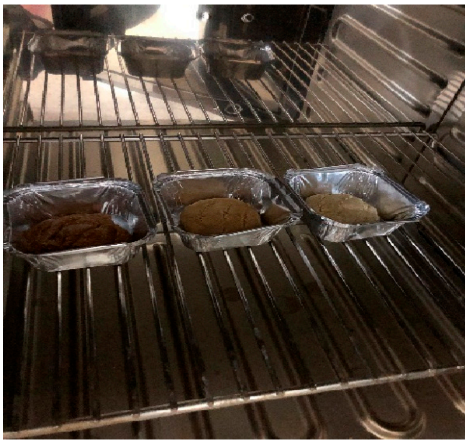

Figure S4. Bread during fermentation, showing significant volume increase.

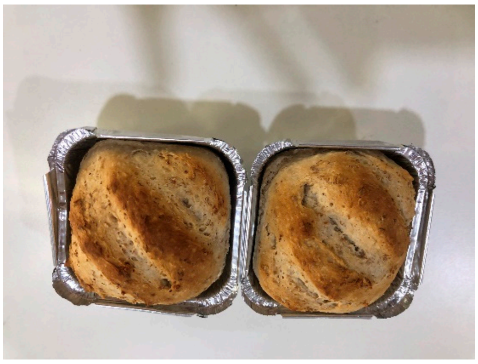

Figure S5. Sensory Analysis Sheet for Bread Evaluation

Date: Tester N°:

Gender: Age:

Bread sample:

	1	2	3	4	5	6	7
Volume	☐	☐	☐	☐	☐	☐	☐
Color	☐	☐	☐	☐	☐	☐	☐
**Alveolation**	☐	☐	☐	☐	☐	☐	☐
Taste	☐	☐	☐	☐	☐	☐	☐
Aroma	☐	☐	☐	☐	☐	☐	☐
**Crispness**	☐	☐	☐	☐	☐	☐	☐

	1	2	3	4	5	6	7
**Hardness**							

Figure S6. Inhibition curve of BP-Mc (30%) by DPPH assay

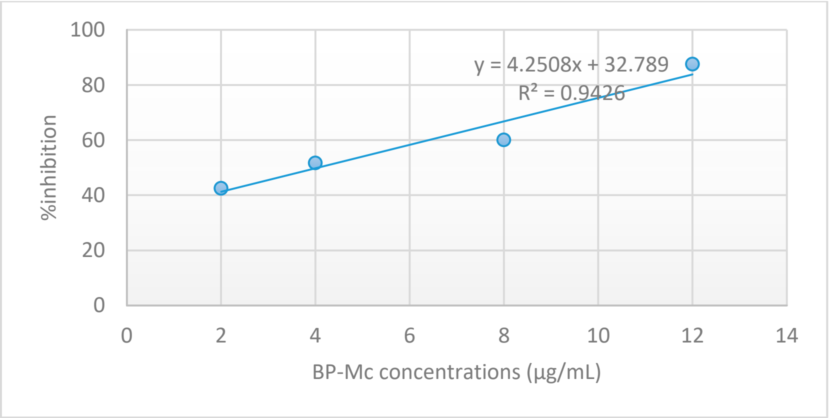

The inhibition curve shows the scavenging activity of BP-Mc (30%) at varying concentrations in the DPPH assay. The percentage of inhibition was calculated based on the reduction in absorbance. The results are expressed as % inhibition relative to the control.

Figure S7. Inhibition curve of BI-Mc (30%) by DPPH assay

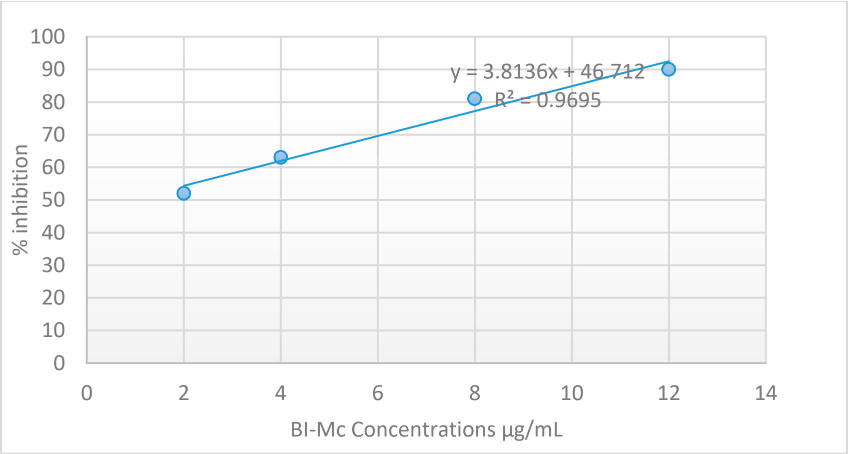

The inhibition curve shows the scavenging activity of BI-Mc (30%) at varying concentrations in the DPPH assay. The percentage of inhibition was calculated based on the reduction in absorbance. The results are expressed as % inhibition relative to the control.
